# A Computational Model for Collective Cellular Motion in Three Dimensions: General Framework and Case Study for Cell Pair Dynamics

**DOI:** 10.1371/journal.pone.0059249

**Published:** 2013-03-19

**Authors:** Federico Frascoli, Barry D. Hughes, Muhammad H. Zaman, Kerry A. Landman

**Affiliations:** 1 Department of Mathematics and Statistics, University of Melbourne, Victoria, Australia; 2 Department of Mathematics and Statistics, University of Melbourne, Victoria, Australia; 3 Department of Biomedical Engineering, Boston University, Boston, Massachusetts, United States of America; 4 Department of Mathematics and Statistics, University of Melbourne, Victoria, Australia; Centrum Wiskunde & Informatica (CWI) & Netherlands Institute for Systems Biology, The Netherlands

## Abstract

Cell migration in healthy and diseased systems is a combination of single and collective cell motion. While single cell motion has received considerable attention, our understanding of collective cell motion remains elusive. A new computational framework for the migration of groups of cells in three dimensions is presented, which focuses on the forces acting at the microscopic scale and the interactions between cells and their extracellular matrix (ECM) environment. Cell-cell adhesion, resistance due to the ECM and the factors regulating the propulsion of each cell through the matrix are considered. In particular, our approach emphasizes the role of receptors that mediate cell-cell and cell-matrix interactions, and examines how variation in their properties induces changes in cellular motion. As an important case study, we analyze two interacting cells. Our results show that the dynamics of cell pairs depends on the magnitude and the stochastic nature of the forces. Stronger intercellular stability is generally promoted by surface receptors that move. We also demonstrate that matrix resistance, cellular stiffness and intensity of adhesion contribute to migration behaviors in different ways, with memory effects present that can alter pair motility. If adhesion weakens with time, our findings show that cell pair break-up depends strongly on the way cells interact with the matrix. Finally, the motility for cells in a larger cluster (size 50 cells) is examined to illustrate the full capabilities of the model and to stress the role of cellular pairs in complex cellular structures. Overall, our framework shows how properties of cells and their environment influence the stability and motility of cellular assemblies. This is an important step in the advancement of the understanding of collective motility, and can contribute to knowledge of complex biological processes involving migration, aggregation and detachment of cells in healthy and diseased systems.

## Introduction

Cell migration is a fundamental phenomenon throughout all the stages of animal life, from its commencement to its end. Cells may move as individuals, in several distinct ways, or may move collectively as chains, clusters or sheets. A variety of complex mechanisms govern these motions in contexts as different as embryonic morphogenesis, wound healing and cancer development [1], [2]. The last case is one of the most investigated examples in the literature, with the use of computational and analytical models focusing on aspects such as the growth of masses of tumor cells, the importance of blood and nutrients on their development, and the shapes of different cancer types [3]–[6]. Experimental evidence suggests that quantitative models have the potential to capture the mechanisms in cellular motility realistically and faithfully [7].

From a biophysical point of view, although factors affecting motion of single cells are beginning to be understood [2], [8], still little is known about motion when cells are in groups. In particular, understanding the mechanisms that favor collective migration over movement in isolation constitutes a major challenge [9], and a number of approaches have been developed. Well-known contributions are, for example, those by Drasdo and others [10], [11], which describe the dynamics of tumor formation using an off-lattice framework, proliferation and intercellular forces, or those by Glazier et al. [12], [13], who use aggregation on lattices via cellular Potts models. Other examples are given by cellular automata for a stochastic description of solid tumors [14], continuous formulations [15], [16], reaction-diffusion type equations [17], dissipative particle dynamics [18] and the use of methods inspired by molecular dynamics [19]. Similarly, but in the context of two-dimensional motility, a number of analogous paradigms are used to describe the way cells move to close wounds or grow tissue [20]–[24].

Together with theoretical developments, experimental advances in the last few years have also been substantial, especially with regards to the measurement of forces acting on cells and on cellular surroundings [25], [26]. Examples for monolayers of epithelial cells are established [27]–[29], and measures of collective activity that have the potential to inspire fundamental theoretical modeling have also been provided [30]–[33]. Recently, the focus has shifted from two- to three-dimensional movement, either for isolated cells [34], [35], and for groups [36]. These studies emphasize the importance of considering the distribution of forces across cell surfaces and the dynamic interactions between cells, their neighbors and the external environment for describing cell motion in biological tissue. This is particularly relevant in three-dimensional settings.

It has to be noted that, while not without interest, studies of cell movement on synthetic, two-dimensional substrates inevitably present limited relevance to developmental biology. In fact, motion in vivo usually takes place in a three-dimensional environment and in the presence of significant amounts of extracellular matrix (ECM), which is the complex medium that surrounds cells and with which they interact 37–39. For these reasons, in this work we develop a new model for migration of groups of cells in three dimensions, where the focus is on cell-cell and cell-ECM forces. This framework pays particular attention on the role of cellular receptors mediating cell-cell adhesion and cell-ECM traction forces, and their properties and dynamics.

## Materials and Methods

Living cells exist in a variety of different shapes, most of which are also subject to variations according to a number of internal and external factors. Examples are given by the forces that neighboring cells exert on each other, the interactions with the surrounding matrix and by cellular programs that change the properties of cells or of its parts.

As with other well-established models in the literature [40], in our model cells are nominally spheres with a constant given radius, and are allowed to overlap partially to account for the deformability that biological cells possess. A novel feature of our method is that each sphere is provided with sites on its surface, representing coarse-grained cellular receptors: these exemplify collections of the contacts mediating the essential interactions among cells and the surrounding environment. This approach is inspired by well-established models present in the literature, which are able to capture properties of cell-cell or cell-ECM interaction [41], [42]. There are two different types of receptors in this model: the cell-cell adhesion sites (C-C) and the cell-matrix ones (C-M). The former are responsible for the adhesive forces acting among different cells, the latter mimic the attachment, adhesion and detachment process of cells when propelling through the ECM. Sites are randomly assigned on each cell surface at the beginning of each simulation, according to a uniform distribution. They can also be re-allocated at given times. In this work, we do not consider the possibility of receptors continuously migrating on cellular surfaces or their numbers being varied in the course of a simulation run.

It is assumed that cellular propulsion through the ECM occurs through four different stages, that take place during each integration step. This mechanisms are given by: a protrusion phase by which the cell searches for available ligands in the ECM, an attachment stage in which the cell grabs the available ligands, and finally traction and detachment phases through which the cell advances through the matrix and dislocates itself from previously held ligands. For simplicity, we assume that protrusion and traction forces occur along the same direction, and neglect the effects of the ECM attachment and detachment on cell dynamics.

The ECM is a complex medium with a heterogenous structure, through which cells can display a rich variety of biological behaviors, which in turn have nontrivial effects on their motility. As a first approximation, the model encapsulates the resistance that the ECM exerts on cells by considering the matrix as a viscous, homogeneously distributed medium, with a constant viscosity 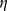 whose value does not change with time or space. Cell-ECM interactions also do not induce any deformation effects on the cellular surface, or modify the structure of the ECM. For example, ligands are assumed to not deplete as cells move through the medium.

As with previous modeling of single-cell motility [43] and cellular approaches to collective motion [10], [40], inertial effects are ignored, so that cell-cell and cell-ECM interactions are balanced at each cycle:
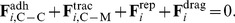
(1)


The term 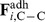 describes the total adhesion force experienced by cell 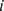 via its C-C sites, which act according to an attractive term depending on the distances of similar sites of neighboring cells. The term 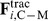 expresses the traction force that pushes each cell forward in the ECM, and is the resultant of the radially outward forces that are generated by the ECM sites on the surface of cell 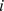. The term 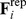 is the repulsion between 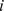 and the cells that are overlapping it, and captures the small deformability of cell 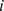 [44]. Finally, 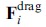 describes the effect of resistance due to the ECM on cellular motion: resistance is assumed to be linear in velocity and expressed as a Stokes term, but this particular interpretation is not essential to the model. The term reads
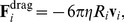
(2)where 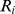 and 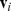 are the radius and velocity of cell 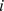. The factor 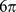, appropriate for a sphere of radius 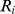, is of the same order of magnitude for other compact shapes of nominal radius 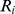.

By substituting the above equation into Eq.(1) and solving for the velocity, the equation of motion for a cell 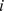 is obtained:
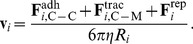
(3)


By solving this equation for a given timestep 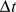, we have a description of cellular motility for interacting groups of cells, which depends on the way forces are modeled.

From now on, we adopt the convention that *subscript indices refer to cells*, while *superscript indices refer to sites*.

### Adhesion Force Mediated by Cellular Sites

Cell-cell adhesion is known to be mediated by cadherin molecules and, depending on their types, energies among receptors as functions of their distances have been, to some degree, experimentally measured. Generally, energy profiles are characterized by a binding negative minimum and a positive, repelling contribution when receptors are pushed close to each other [44]–[47]. Adhesive bonds are ultimately responsible for the mutual pull that cells experience when they are sufficiently close. Also, as the contact area between cells increases as a result of this pulling action, there is a consequent increase in the number of sites that engage, with a further growth in the adhesive force. This effect must be counterbalanced by the limited deformation that cells are able to sustain, for their structural properties and steric effects.

The attractive part of cell-cell interaction is expressed by 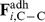, which is the resultant of all the forces acting on cell’s C-C sites. For the reasons discussed above, adhesive interactions are treated differently if sites are in the overlapping area between cells or not. So, in our model, adhesion can have two different expressions, depending on the location of sites with respect to the mutual position of the cells: one contribution when sites are outside the common area between overlapping cells and another one when they fall inside that area.

For the first case, consider two sites 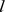 and 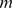 belonging to two different neighboring cells 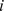 and 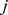 respectively. If the distance between cell centers is 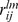 and the sites are not in the overlapping area between the two cells, the force acting on site 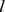 due to site 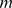 is given by
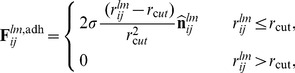
(4)where 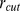 is the largest distance for which the C-C force acts. For 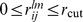 this force is a linear function of distance between the cell centers, and acts along the line of the two sites 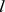, 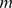 given by the unit vector 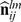. The force depends on 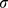, i.e. the energy scale of the contact-making process between sites. For distances larger than 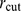, the force 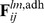 is zero and sites are not able to pull cells closer. Observe that adhesion force pulls engaged receptors together with an increasing strength as their distance is decreasing, in line with theoretical and experimental findings [40].

**Figure 1:**
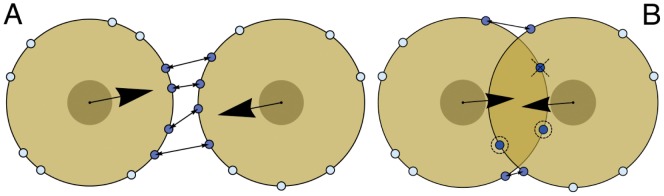
Representation of adhesion forces. A two-dimensional sketch of two typical scenarios for the evaluation of cell-cell adhesion between two cells is presented. In (A), cells have no shared surface area, in (B) cells have a shared surface area and sites are present in both overlapping and non-overlapping regions. Sites colored in light blue do not interact, because their distance from available, non-engaged neighboring sites is larger than 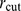. In (A), sites in non-overlapping regions act one-to-one, so that bonds do not cross. When cells overlap partially, as in (B), only pairs of sites trapped in the shared surface area are considered: two sites, each from a different cell, are engaged and circled; one unpaired site is crossed and inactive. In (B), sites in the non-overlapping area close to the shared surface of cells are also engaged. Their contribution is summed to that coming from the two sites in the overlapping region, so that the total adhesion force is obtained. In both scenarios, the total force from all engaged sites is applied to the center of the cell.

As shown in Fig. 1, we require that C-C sites always interact in a one-to-one manner: at each time, only one site 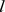 from cell 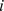 can be engaged with another site 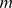 of cell 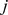. Among all possible choices for 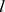, i.e. among all the sites of cell 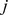 that are at a distance less than 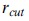 from 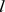, the site 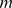 at the smallest distance is preferred. This prevents as much as possible the occurrence of cross-interaction of bonds between engaged sites. The case when bonds traverse each other is extremely rare, or, in other words, the directions in which C-C adhesion forces of adjacent pairs of sites are applied are very unlikely to cross. To enforce this, numbers that identify sites in each cell are randomly permuted at each timestep, so that the order in which sites are selected for the negotiation of the one-to-one action is changed. This implies that site interactions are also recalculated, and negotiation starts anew at each 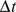. Also, it is important to realize that, for geometrical reasons, crossing between two C-C adhesion force vectors in a three dimensional space is uncommon. During model validation, no occurrence of cross-interaction has ever been registered.

All this aims to reproduce the interaction among cellular contacts in nature, where cadherin is exchanged among two receptors from different cells and no crossing between them is permitted [48]. Notice, however, that the C-C sites in this model are a coarse-grained version of real receptors, and are thus intended as means of modeling the effective cell-cell interactions for the timescale assumed in this framework. We will elaborate further on this point shortly.

The second scenario for adhesive interaction among cells occurs when cells overlap and C-C sites fall inside the overlapping area. In this case, the force is assumed to depend in a simple way on the number of sites shared between the two cells. If 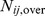 represents the number of sites of cell 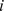 trapped in the overlapping region between cell 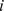 and 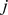, and, similarly, sites of cell 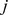 enclosed in the same area are given by 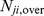, we define 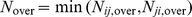 and write
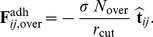
(5)


This force acts along the line of centres of cells 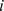 and 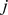, given by the unit vector 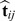, and depends on the energy scale 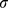. It is assumed that only the minimum number of the overlapping sites for cell 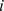 and 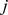 participates, because sites need to be paired for exerting attraction onto each other. This choice is also consistent with the viewpoint adopted for C-C site interactions outside the overlapping area.

The term that contains the total adhesion force on cell 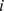 due to cell 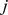 is a sum of Eq.(4) for C-C sites that are outside the overlapping area and Eq.(5) for sites trapped in regions shared between cells. If 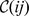 indicates the set of interacting C-C sites for cells 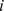 and 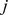 in the non-overlapping region, we can write
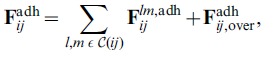
(6)where the sum is over all pairs of sites of cell 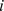 and 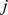 that are engaged according to the one-to-one rule. In Fig. 1(A), 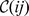 contains a total of eight C-C sites, four for each cell. The first term in the RHS of Eq.(6) is constituted by the four contributions associated to the one-to-one interactions among pairs of sites. The second term in Eq.(6) is instead zero, because cells do not overlap. In Fig. 1(B), 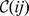 contains four C-C sites in the non-overlapping regions of the cells, two for each cell. Also, in this case, there is a further contribution coming from the sites inside the shared area, so that both terms in Eq.(6) are non-zero.

If we consider more than two cells and cells adhere to more than one neighbor, the term representing the adhesion force 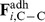 in Eq.(3) for cell 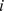 is the sum of terms like Eq.(6) for all the cells that surround 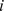, whose sites are correctly paired to 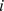
’s sites. This force depends on the distribution of sites and is stochastic if the sites are re-assigned at each timestep.

Note that all forces in this model are applied at the center of the cell. For adhesion, in a typical simulation run we determine which sites are engaged and which ones are overlapping, calculate the forces due to each pair and apply them to the center of the cell being considered. In this way, the term 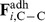 is obtained: after all other forces are calculated, Eq.(3) can be solved for the velocity 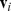 of the cell.

### Traction Force and Surface Sites

Traction force originates from interactions between cells and the surrounding matrix, and is mediated by sites on the surface of model spheres of a different type from the adhesive ones discussed above. There are two fundamental characteristics of these C-M traction sites: they are active only when not trapped within the overlapping region among cells, and they produce a radial, outward pointing force intended to capture the way cells propel themselves through the ECM. Two of the model assumptions are that the ligands in the ECM are unlimited and that no exposed C-M is inactive, i.e. not exerting any traction. Each of the C-M sites is thus considered to produce a contribution to force at each timestep 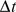, if it is exposed to the ECM.

**Figure 2:**
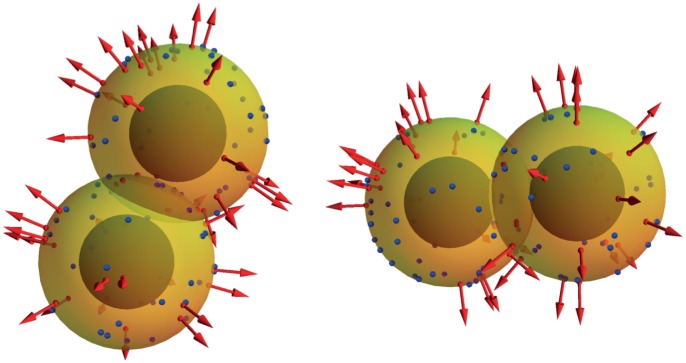
Three dimensional sketches of two interacting cells, from different viewpoints. C-M traction sites are in red, and, when exposed to the matrix, generate radially normal vectors representing traction forces. Note how red sites trapped within the overlapping surface area of cells do not generate any force. C-C adhesion sites are also present on each cell and are shown in blue, whereas the impenetrable cores are depicted in dark grey.

So, assuming that a C-M site 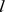 of cell 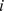 belongs to the cellular surface area that is exposed to the surrounding medium, the site 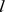 contributes to a force given by
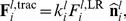
(7)where 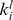 is the adhesivity function of site 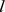, 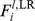 is its force per ligand-receptor complex and 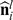 is the normal to the sphere surface at the position where the C-M site 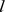 is located. A three dimensional depiction of two cells, C-M sites and traction vectors is sketched in Fig. 2. The biological meaning of these quantities and their experimental accessibility is discussed elsewhere [43].

For simplicity, in the following we assume that 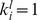 for all sites and cells, and that the force per ligand-receptor complex is constant and equal for every receptor, i.e. 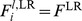. The traction force acting on one cell at each timestep is the resultant of the single contributions described by the previous equation:
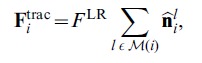
(8)where 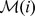 is the set of interacting C-M sites, i.e. those sites of cell 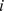 that are exposed to the matrix. This force depends on the distribution of sites and is stochastic if the sites are re-assigned at each timestep. Note that if the sites are non-randomly but uniformly distributed over all the sphere surface and their number is such that the entirety of the surface is covered, if the two spheres do not overlap then the total traction force on each sphere is zero. In this work, any cells in isolation that are completely exposed to the matrix always display a non-zero total traction force because the sites are randomly chosen and are limited in number.

### Repulsion Force

When the nominal spheres representing cells overlap, a repulsive force comes into play. This embeds in the model the energy cost to a cell incurred on deformation. When overlap occurs, we take the repulsive force to be a linear function of the distance between the cell centers, cut off by an infinite wall if the distance between cell centers falls below a prescribed minimum:
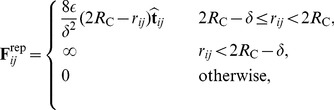
(9)where  is the energy scale for cell-cell repulsion, 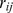 is the distance between cells 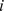 and 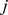, 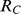 is the (fixed) cell radius, 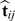 is the unit vector between the centers, and each cell possesses an incompressible, impenetrable core with diameter 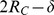. From now on, we express 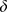 in terms of cell structural softness by introducing the compressibility 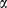, so that 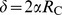.

This treatment is similar in some respects to existing models of cellular dynamics [10], [18], [19], but differs in important ways. In the previous works, either the effects of interactions between cells are incorporated into a single deterministic force term, combining cell-cell repulsion with cell-cell adhesion, or a limited number of sticky sites that are not allowed to overlap are used. In our model, emphasis is placed on cellular contacts and their dynamics instead, giving the repulsive and adhesive forces a significant level of stochasticity and capturing some attributes of the interaction at finer scales than in a purely continuum treatment. This has important consequences for the motility of groups of cells, and it will be amply discussed when our findings on cellular pairs are presented.

### Further Considerations and Model Assumptions

Our model contains relevant innovations that are motivated by experimental results. It combines a stochastic treatment of cellular movement via surface sites with inhomogeneous and localized adhesion via cell-cell sites. Rules used for C-C sites negotiation and C-M sites interactions are inspired by the dynamics observed in the laboratory. Deformations resulting from cell-cell mutual push and pull effects are also considered. This framework is thus capable of quantitative estimations for a number of measurable observables for three-dimensional arrangements of cells, and findings for the dynamics of pairs will be shown in the next section.

We now describe a typical simulation run and comment on the approximations introduced. At the initial time 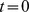, the C-C and C-M sites are assigned on each cell. The numbers of sites 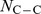 and 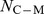 are the same and stay constant for every successive time 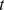. The velocities 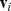 in Eq.(3) can be computed in terms of cell positions at any instant, since, as observed, forces generated by sites are applied at the center of the cell. Note that this also implies that cells cannot rotate about their centers.

In this way, our system consists of a set of coupled, autonomous, first-order, nonlinear ordinary differential equations (ODEs) for the positions of cell centers. At each time step 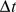, these ODEs are solved by a fourth-order Runge-Kutta scheme, with forces calculated according to expressions previously discussed. The timestep must be chosen according to the nature of the interactions and their biological significance. In line with previous studies [43], it is assumed that, at each 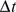, cells have completed their four stages of protrusion, attachment to ligands, traction and detachment from the ECM. This means that the forces that act on any cell at each timestep are not instantaneous, but rather represent the average (or effective) value of each of the different types of forces within each cell movement cycle.

Clearly, different timescales are present in cellular interactions. For example, adhesive bonds among receptors are created and destroyed over scales different than those relevant to deformation effects on cellular surfaces or the resistance due to cell-matrix interaction. On the contrary, the typical timescale for the movement steps in a variety of cells such as fibroblasts, epithelial and embryonic cells is about 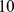 minutes, or 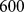 seconds, and this is the value the model uses for 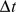 [21].

Movement cycles are generally not synchronous between cells, and do not occur exactly at a given time interval. This means that, when collections of cells are considered, one cell does not complete its movement cycle according to those of others in its vicinity. Determining which factors influence motility steps of protrusion, attachment, traction and detachment in assemblies of cells is a very profound question, but appears of great complexity, both theoretically and experimentally. Asynchronous cells could be incorporated directly in the model, for example by introducing some randomness in the way C-M sites produce traction forces. For instance, each cell could have a probability 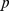 that at each time 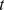 their traction is zero. This would have the effect of introducing delays in the way cells propel through the matrix: at some timesteps only a subset of cells would produce traction and move to different positions within the matrix. As a first approximation, it is *assumed* that cells are *synchronous* and that their movement cycles have all *equal* duration 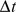, which is the timestep chosen for the integration, as in [43].

As stated, forces are all applied at the center of the cell and not at the surface or where C-C and C-M sites lie. Deformation effects that cells experience as a result of their mutual interaction are not taken into account. One can argue that, in one cycle of duration 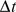, those effects are not of primary significance and can be neglected. Also, having the point of application of all the forces in the cell centers is the simplest approach to take, by which forces are rigidly translated from the periphery to the center of the cell. Deformation effects due to cell-cell interactions are instead important and are captured through the repulsive term 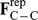. While surface sites involved in C-C interactions generate effective forces only when associated to sites in different cells, surface sites involved in C-M interactions are always effective.

The number of C-C and C-M sites used in the model does not correspond to the biological number of receptors that are present in real cells. These are of the order of 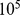
–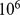 and cannot be represented computationally. As anticipated, a coarse-graining procedure is adopted and the number of sites used throughout this work is given by 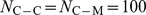. This constitutes a practical compromise between computational feasibility and realistic granularity in cell-cell adhesion and cell-ECM traction forces. Note that, for a sufficiently large number of C-C sites 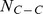, the percentage of engaged and overlapping sites per cell, in the case of cellular pairs, oscillates between 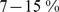. This implies that, for example, simulations at 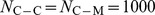 produce similar overall dynamics to those for 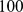 sites for coupled cells, as confirmed when validating the code. Therefore, 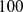 sites ensure that spheres are adequately covered and the distributions of sites are never unrealistically inhomogeneous or too few. Using only 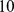 sites, for example, would not allow a realistic description of cell-cell adhesion or cell-ECM traction. Note also that the scales adopted for C-C site interaction 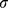 and 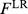 depend on the number of sites considered, given that the adhesion and traction forces that act on cells are the resultant of the interactions among sites. Table 1 contains the values used in this study, which are within well-established experimental ranges.

**Table 1:** Typical non-dimensional values for model parameters.

Parameter	Symbol	Value
Cell Radius	*R_C_*	1
Integration timestep	Δ*t*	0.01
Number of C-C sites	*N*_C–C_	100
Number of C-M sites	*N*_C–M_	100
ECM viscosity	*η*	0.0016
Cutoff radius	*r*_cut_	1
Adhesion Energy scale	*σ*	0.004
Repulsive Energy scale	ò	0.025
Compressibility factor	*α*	0.5
Adhesivity Function	*K*	1
Force per ligand/receptor complex	*F* ^LR^	0.01

In real units, we have 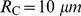, 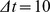 min, 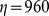 P, 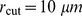, 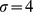
×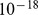 J, 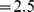
×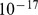 J, 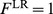 pN. Note that the choice of 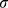 for a total number of C-C sites 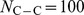 is in the order of the typical cell-cell adhesion energy for a single cell, i.e. 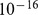 J [40], [54].

The C-M sites can be randomly relocated on cellular surfaces during the simulation runs or can remain fixed in their initial position at 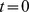 for every subsequent 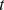. The first case is a good representation of the dynamics of receptors during the stages of cellular movement, where they rearrange themselves along the cellular surface to probe the surrounding media in search of ligands. We call this the *dynamic* regime. In this case, the ordinary differential Eq.(3) becomes a stochastic differential equation: the same Runge-Kutta scheme is used and the site positions are not randomly updated for the duration of integration between 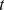 and 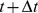 [10], [49]. Hence, stochastic rearrangement of sites occurs at each timestep and not between timesteps; note that this approximation is in line with the time resolution that is experimentally available. The second, static case can be interpreted instead as an abstraction for the so-called *persistent* regime [50], where cells tend to maintain a specific direction of propulsion through the matrix. One example when this behavior arises is given by the presence of a chemotactic gradient in the ECM, whose overall action is to guide cells towards regions at higher concentrations.

Similarly, cell-cell adhesive sites can move along the cell surface. In reality, two mechanisms are responsible for C-C site migration on the cell: shuttling (or internalization) and diffusion within the cellular membrane [51]. Specific modeling of these two processes is not included and goes beyond the scope of the present work. Once C-C sites of different cells engage, it is reasonable to claim that only a small amount of site movement occurs, since the cells change shape slowly and with displacements that are negligible on the time scale given by a movement cycle 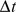. In the Results and Discussion section, the cases when C-C sites are kept fixed and when they are randomly shifted on the cellular surface at each timestep are discussed and compared. Although the biological processes of cadherin receptor migration is more complex, our approach sheds light on the role of adhesive sites on cell-cell interactions.

Finally, a further approximation contained in this work is the assumption that the ECM is homogeneous, and that its resistant action on the cells can be expressed through Eq. (2). This is in line with similar treatments in the literature [10], [52], although local variations of the rheological properties of the ECM and the effect of the geometry of cell clusters on transport cannot be fully captured. Nonetheless, the use of a Stokesian term can still be reasonable, since one can consider that inhomogeneous contributions have been averaged out for the duration 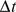 of cell movement stages of attachment, protrusion, detachment and traction.

### Parameter Values

We will consider the dynamics of cell pairs and also a large cellular cluster made of 50 cells.

At the beginning of each simulation a number of C-C and C-M sites are randomly assigned on each cell, i.e. 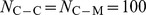. Cells are at 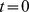 at a fixed center-to-center distance, which is, at this stage in our study, given by 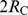 (i.e. cells touching). Other initial separations will be considered later.

Typical values for the model parameters are shown in Table 1 in non-dimensional units. If fundamental time, force and length scales are indicated by 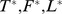, Eq.(3) can be normalized and all quantities expressed as functions of these. In our case, 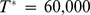 s (or approximately 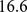 hours), 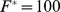 pN and 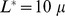
m are chosen.

Cell radius is assumed to be 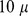
m, and resistance to the ECM is modeled with a choice of viscosity 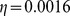, corresponding to 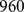 P, which is a common experimental value for ECM in the literature [53]. Adhesion, traction and repulsion scales are chosen to match the experimental ranges reported in the literature, and, in the case of 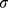 and 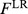, are adjusted to our choice of having 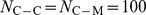 [54], [55]. Cellular compressibility is 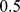, or half a cell radius, and the effects of softness and other intrinsic properties of cells on cellular dynamics will be one of the objects of discussion of our analysis. The cutoff radius is chosen to be equal to the cellular radius, so that cells can engage C-C sites when in proximity, similarly to Ref. [19]. This is a good compromise: a 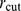 which is too large is not biologically realistic, whereas a 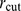 which is too small increases the adhesion force [Eq.(6)] and causes the spheres to have overlapping compressive cores, thereby preventing the formation of stable bound states.

Different combinations of 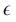, 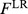 and 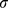 can result in similar dynamics, since the total force can be the same for a large set of adhesion, repulsion and traction parameters (see Eq.(3)). Control values for 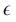 and 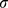 are chosen in the following way: starting from an equilibrium condition with no traction force (i.e. with 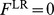), values that allow cells to form a bound state are selected. By varying 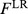, it will be possible to check how stable the pair is when traction increases.

With parameters in Table 1, we observe average forces in the ranges of 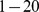 pN per cell, with adhesion generally stronger than repulsion. The average velocity of cells when they are in a steady state usually varies between 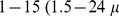
m/hr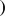, and it can sometimes be higher when cells are relaxing towards a bound state, i.e 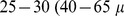
m/hr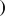. These ranges are realistic and consistent with experimental results [43], [56], [57].

## Results and Discussion

An important case study of cellular pairs is now considered. This allows us to discuss the capabilities of our model in detail and represents conditions that are experimentally important. In fact, cellular pairs are frequently present during in vitro experiments, for instance as a result of the disaggregation of large cellular clusters or as the first stages of complex aggregation mechanisms [57], [58].

### Dynamics of Cell Pairs

The goal of our analysis is to capture the stability of pairs with respect to the individual properties of their members. First, we investigate how two cells interact by varying 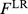, i.e. the magnitude of individual traction for each C-M site. A growing 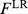 represents changes in the surrounding ECM that cause an increase in the traction force, for example for the presence of a higher concentration of ligands that alter cellular motility. The traction magnitude is kept constant for all 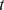. We shall explore the two major alternate cases for pair dynamics: a bound or steady state, where cells are travelling together at a center-to-center distance that is equal or less than 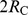, and a detached state where they form no link, are in isolation and their mutual distance is larger than twice the cell radius.

If the number of C-C and C-M sites and the repulsion energy scale are fixed, two cells display different behaviors for different values of traction forces. The two factors influencing their dynamics are mainly given by the strength of adhesion interaction and the dynamics of sites on the cell, i.e. whether they are allowed to be randomly updated at each timestep or not.

It is useful first to look at typical steady and detached states for four combinations of receptor dynamics, since they capture a variety of interesting scenarios. Case 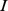 is defined as *persistence* and corresponds to C-C and C-M sites being fixed for all 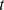, capturing the effects of a chemotactic gradient. Case 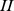 has C-C sites fixed and C-M receptors randomly updated at each timestep, mimicking the migration of traction sites occurring during the stages of cellular movement through the ECM (protrusion, attachment, traction and detachment). Other possible combinations for rearranging sites are also considered: case 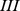, where C-C sites are varied and C-M sites are fixed, and case 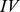 where both types are randomly updated. These conditions are summarized in Table 2.

**Table 2:** Rules for site allocation.

	Case I	Case II	Case III	Case IV
C-C sites	Static	Static	Dynamic	Dynamic
C-M sites	Static	Dynamic	Static	Dynamic

Rules used for the allocation of sites in the four different cases discussed in the text. *Static* indicates that sites are not changing position as time evolves, whereas *dynamic* means that sites position is randomly reallocated at each timestep and for each site of the chosen category (i.e. C-C or C-M sites).

**Figure 3:**
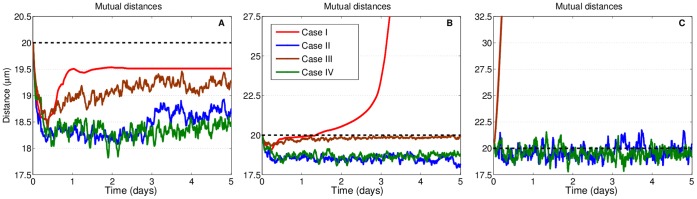
States for two interacting cells. Distance between cell centers versus time for three values of traction per site strength 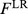. Typical examples of bound and detached states for a pair of cells with C-C and C-M sites with different dynamics. The dashed line indicates the distance at which cells are tangent (center-to-center distance is twice the cell radius), representing the border between a bound and a detached state. (A) 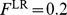 pN, with bound states. (B) 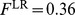 pN, with detached states for the persistent case and bound states for the remaining cases. Note the three regimes for case 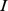, as discussed in the text. When isolated, cells for case 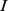 advance linearly because of Eq.(3). (C) 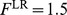 pN, with detached states for cases 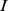 and 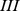 (with almost identical curves) and combinations of bound and detached states for cases 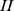 and 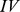. Legend for all three examples is in (B). Note the different scales for the 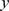-axes in the three plots.

Figure 3 illustrates the states of the cellular pair over the total simulation time of 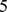 days, for three values of the traction magnitude per site 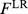, and a fixed value of adhesion energy per site is 
×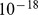 J.

Examples of bound states are presented in Fig. 3(A), The initial distribution of sites is the same for all four cases, which develop differently in time depending on the rules for the site dynamics. Case 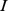 (in red) shows an initial phase where distance decreases to a minimum due to the adhesive force among C-C sites, then increases up to a local maximum for a rebound caused by a dominating repulsion and finally exhibits a damped oscillation towards a steady state after about 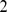 days. The pair has reached an equilibrium so that the total force experienced by each cell is constant and the center of mass moves at constant velocity, in the direction of the vectorial sum of the total forces for each cell. At times preceding equilibrium, cells are moving around each other and being pulled closer by C-C sites, which bind together depending on their mutual position. In this case, cells are able to arrange together so that a stable configuration is reached and they can propel in an attached state through the ECM.

Case 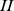 (in blue) displays a lower intercellular distance than persistent case 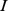, and, after following a similar trough for the initial half day, it remains at a lower, almost constant distance for the rest of the run. The effect of changing C-M sites at each timestep is evident by the oscillatory character of the trajectory. This is also true for remaining cases 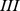 and 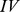. Varying only C-C sites produces a smaller effect, with respect to persistence, than changing C-M sites only or C-C and C-M together. If the direction of traction force changes constantly and randomly as a result of reassigning C-M sites at each 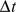, adhesion between cells is more effective overall, because traction will be less frequently in a direction opposed or unfavourable to adhesion. This is confirmed by the fact that, in case 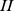, the depth of the initial trough is deeper than that of case 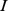, and the pair distance remains lower in case 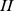 for all subsequent times. In this case, cells will be closer together overall. This is an important point and it will become even more evident in the next Section, when we discuss the behavior of pairs as a function of traction force from a statistical point of view.

Variation of adhesion sites results in a lower stabilizing effect than case 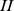, and pair distance is still less than observed in the persistence case, but only slightly. The reason is that C-C sites are reassigned randomly at each timestep, and this affects both sites that are interacting and those that are not. On average, and for a sufficiently large 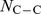, the reassignment causes a larger number of C-C sites to interact than simple persistence, either externally and in the overlapping area. Changing both types of sites has an effect that is very similar to only changing C-M sites, and whether case 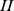 or 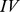 pushes cells closer seems just a matter of chance.

When the traction intensity is increased while keeping a constant adhesion intensity, cells do not necessarily stay together. Fig. 3(B) shows results for the four cases when 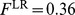 pN, which causes the cells in the persistent type of run to detach. Three different regimes can be identified for case 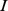: at the beginning and up to 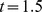 days, the pair shows a bound state as in the previous figure but, given the higher 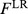, its distance increases and cells lose contact. Approximately between 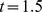 days and 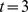 days, their reciprocal separation enlarges in a nonlinear fashion, due to the fact that C-C sites are inhomogeneously placed on the cells and the adhesion attraction between them is still actively counteracting the traction push. After all sites are beyond the cutoff distance, around 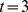 days, cells spread out in the ECM with a larger linear velocity. Between these last two regimes the curve shows an increasing slope, indicating that cells are accelerating with respect to each other due to the progressive decrease in adhesion. This slope becomes steeper as time progresses from 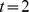 and 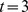 days: this is a combined effect due to the lesser number of sites being engaged and the adhesion force in Eq.(4) decreasing as distance between paired sites increases.

Given the same 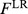, the change from a bound to an unbound state is highly dependent on the distribution of C-C sites and the adhesion scale 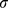. For all cases 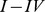, there are situations in which cells detach and then reattach because the adhesion is sufficiently strong and the spatial organization of sites is favorable. Similarly, the permanence in an unbound state where sites are still engaged can vary, as well as the accelerating state before final, independent motion. In the example in Fig. 3(B), cases 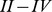 do not split, and show analogous features as in Fig. 3(A). As observed, allowing sites to redistribute generally increases cellular stability.

If traction becomes very strong, cells always experience unbound states, as illustrated in Fig. 3(C) for 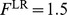 pN. At this value, case 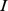 and 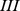 are almost identical: the pull on cells is too large and shuffling C-C sites has no impact at all. Instead, for the remaining cases, cells spend a significant time in separation, but adhesion clearly counteracts random traction more effectively than directed traction. If 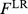 is increased even further, cases 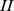 and 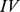 display full unbound states and cells are not able to rejoin. In these cases, and after cells have separated, their mutual distances behave in a typical Brownian fashion (not shown).

### Statistics for Cell Pairs

The fate of pairs is very sensitive to a number of factors: the initial distribution and the updates of C-C and C-M sites, the parameters regulating the intrinsic properties of cells, the forces they are subject to, etc. It is interesting to consider how changes in these parameters shape the dynamics of pairs for a large number of events, so that statistical considerations on the average outcomes for cellular pairs can be made. To do so, we consider results from simulations starting with identical parameter values but different initial distributions of sites.

**Figure 4:**
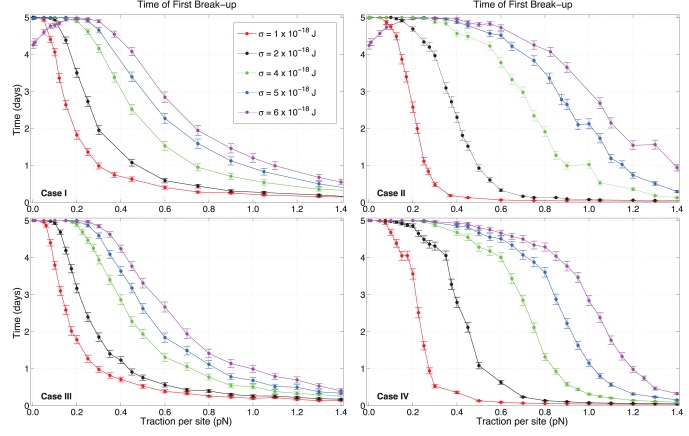
Times of first break-up versus traction per site strength 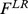 for various values of C-C adhesivity 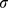. According to Table 2, time of first break-ups for cells when sites are held fixed or rearranged are shown. Each point corresponds to 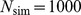 independent runs starting from different randomly generated distributions of sites. Error bars are given as twice the standard error of the mean. Note that, in cases 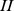 and 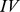, a slower convergence for some specific values of random traction occurs: occasional pairs of points for 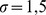 and 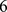
×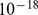 J have similar TFBs for adjacent values of 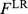.

In Fig. 4, the times of first break-up (TFB) are reported for all four cases and for different values of traction and adhesion scales 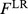 and 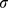 respectively. TFB represents the instant at which the pair finds itself at a mutual distance larger than twice the radius for the very first time, so that the pair experiences its first unbound state. Each point represents the average for 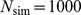 independent runs, where cells at 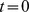 are at a center-to-center distance of 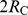. Each run has a different initial distribution of C-C and C-M sites, and error bars are given as twice the standard error of the mean. Different colours identify different values of 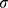.

The plots show similarities between cases 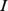 and 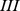, and between cases 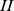 and 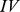. Cases 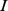 and 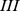 present curves that have mostly overlapping error ranges. For smaller 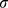, it appears that first break-ups occur slightly quicker for persistence than for runs where C-C sites are reassigned, whereas the situation is reversed for larger adhesion. So, for small adhesions, updating C-C sites results in modestly stabler configurations than persistence, whereas the opposite occurs when large adhesions are acting, i.e. persistence has later TFBs. This is enhanced at very large tractions, where the tails in the curves for cases 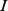 and 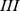 differ the most.

The most significant divergences are observed for cases 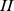 and 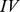, when compared with the other two. The curves show different concavities and updating C-M sites has a major stabilizing effect on simulations. It is interesting to note that cases 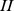 and 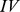 also have quicker, steeper and lower descents towards zero, especially at low adhesions. Variations in TFBs seem more abrupt when traction sites are stochastically reassigned. Also, comparing 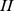 with 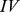 shows that break-ups occur earlier when varying both C-C and C-M sites rather than varying C-M alone. This is especially clear at large values of 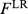, where cases at 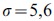
×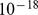 J have tails that are visibly lower and quicker in case 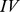 than those in case 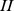. For 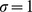
×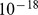 J, the situation is reversed. This once more shows that changing C-C sites has a different effect depending on the adhesive strength the sites can exert on each other.

Generally, varying C-M sites is a stabilizing strategy at small tractions for every adhesion. This can be inferred from the plots, since cases 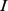 and 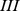 have smaller TFBs than cases 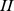 and 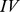. But, as traction increases and depending on the adhesion, there is a point where these two classes of curves intersect, and the situation is reversed. For the smallest adhesions, i.e. 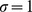
×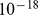 J, this occurs around 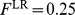 pN. The intersection point between the curves moves towards larger traction values when the adhesion is greater (for example, it is at approximately 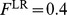 pN for 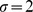
×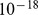 J). When adhesion is sufficiently strong, for instance 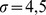
×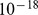 J, cases 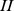 and 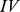 are always more stable than the others. Hence, the way adhesive and propeling forces influence the stability of pairs strongly depends on the dynamics of receptors.

In cases 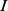 and 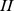, we observe an effect that is not biologically realistic and should be ignored: curves at the highest 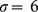
×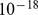 J do not asymptote to the maximum time 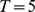 days, unlike the other adhesion scales. At large values of 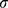, the adhesion force pulls the cells too close together, so that the repulsion 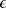 cannot prevent them from having distances less than 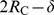. As a result, a large rebound due to the infinite force associated with the incompressible core occurs, and FTBs do not converge to 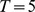. This effect is present only in the two cases where the C-C sites are not updated: C-C site rearrangement avoids the formation of bounds that are too strong and inward directed for too long.

**Figure 5:**
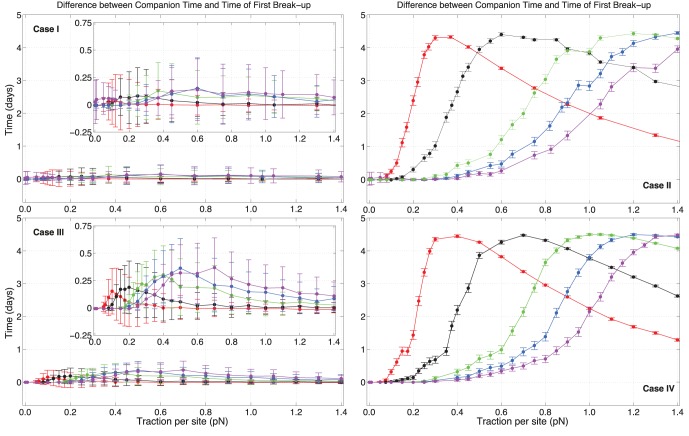
Companion times and times of first break-ups versus traction per site strength 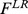 for various values of C-C adhesivity 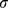. Differences between companion times and times of first break-ups are plotted. When C-M sites are not updated (i.e. cases 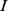 and 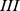), a break-up at time 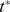 leads to isolated states in the majority of pairs, i.e. cells do not come back together in a bound state for times 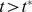. Colors have the same meaning as in Fig. 4, and error bars are twice the standard error of the mean.

To understand how bound states occur in the four cases, we compare the TFBs and the total time cells spend together in each run, which we name the *companion time* (CT). Differences between TFBs and CTs are illustrated in Fig. 5. Persistence entails essentially irreversible break-ups: when detachment begins, it is almost always (in an average sense) driving cells apart and to final isolation. Differences in the inset plot for case 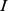 are practically zero within error. The same scenario is repeated for case 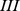, with the presence of some maxima whose position is changing with adhesion values: larger 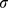 have maxima at larger 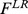. Errors are still large and changing only C-C sites does not give significant variations between CTs and TFBs for case 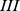 and case 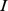.

Completely different plots appear for the remaining cases, which also show very small divergences between them. Visible maxima appear, which move to greater values of traction as adhesion is increased. Extrema are also more peaked for lower values of adhesion. Curves for case 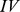 seem to increase with 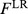 slightly slower than those in case 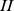, and their rise towards the extrema is more sudden. In general, the higher the difference between TFBs and CTs, the less definitive is the event of a pair break-up, as the cells tend to regroup and still spend a significant part of the simulation time together. As traction gets larger, the difference must decrease since cells are propelled farther and farther from each other and cannot rejoin. A maximum in Fig. 5 represents a transition between the phase at which cells are attached too strongly and do not effectively move together sensibly, and the opposite phase where the propeling force is too large to let them explore the surrounding ECM in a bound state. So, in some sense cells must randomly probe the space around themselves using traction sites if they want to move significantly in the ECM and stay bound at the same time.

**Figure 6:**
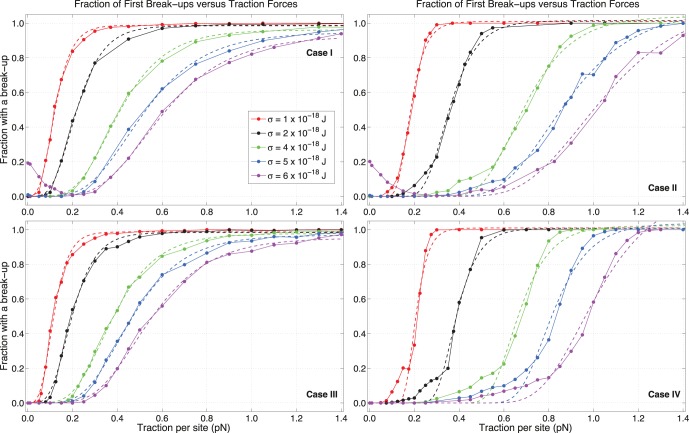
Percentages of pairs with a break-up versus traction per site strength 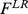 for various values of C-C adhesivity 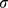. Fraction of simulations that experience a break-up are shown, and fitted with appropriate Gompertz curves (see text). A simpler logistic fit is not as effective, since curves approach the asymptote with different velocities. Data for cases 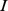 and 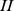 at low traction for 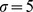
×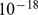 J have not been included in the fit, since they result from rebounds caused by a large, biologically unrealistic overlap among cells.

It is also interesting to consider the percentage of cells that experience a break-up during a run. In Fig. 6, we plot the fraction of runs that have shown at least one such event, and fit them with the Gompertz curve 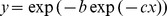, where 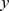 represents the fraction and 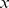 is the value of 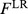. This functional form is chosen because the curves saturate at 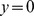 and 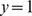 with different velocities. The coefficients 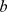 and 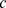 respectively represent the displacement of each curve along the 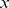-axis and the rate of growth towards the asymptote at 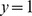. Cases 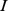 and 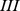 display a good agreement, since they are the cases where randomness has the least effect on the fate of the cellular pairs. Case 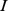 is the best fit of all, showing a generally decreasing coefficient 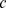 with adhesion and monotonic increase in 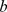 with 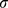. This means that stronger adhesion causes fewer first break-ups (FBs), as expected.

Cases 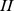 and 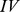 do not follow the Gompertz curve as well. They have a much slower initial rise, a more abrupt jump and steeper ascending phases towards 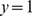 than the previous cases. So, shuffling C-M sites does not only cause later FBs than persistence case 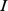 or case 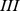, as shown in Fig. 4, but also fewer FBs. Similarly to the analysis of TFBs, differences in the fraction of FBs between cases 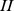 and 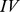 are more evident as adhesion increases and at larger tractions. In particular, reassigning both types of sites causes more break-ups than updating just C-C at large 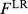 and 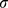.

Until now, we have discussed the variations of pair statistics due to biomechanical parameters, such as forces and site dynamics. We conclude this Section with an investigation of the role of intrinsic cellular properties, such as structural rigidity and resistance to the ECM in the behavior of two cells. This discussion is limited to cases 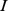 and 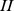 only, because these are the most indicative of all regimes and are the ones that show the largest differences. We maintain a fixed adhesion 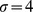
×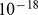 J and consider the effects of varying 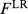 for three different properties: the ECM resistance (changing the viscosity *η*), the initial cell-cell distance 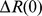, and the cellular compressibility 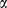 (see Table 1).

**Figure 7:**
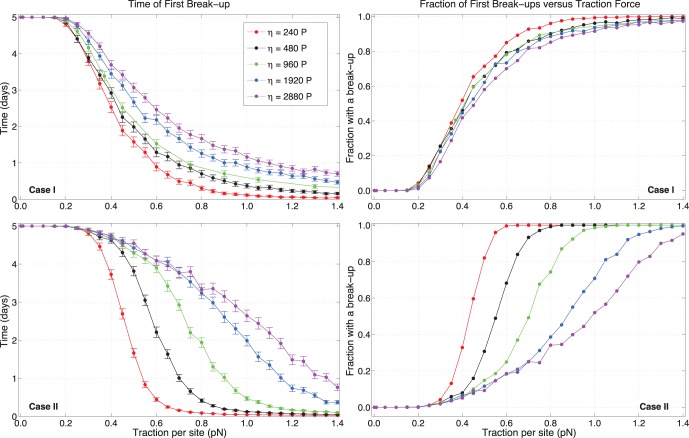
Effect of ECM resistance. Times of first break-up and fractions of runs with a break-up versus traction per site strength 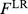, for different values of the ECM resistance (using a linear viscous term 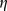). Effects due to stochasticity are evident.

Fig. 7 shows the effects of ECM resistance on pair dynamics, for the cases of persistence (case *I*) and random updates of C-M sites (case 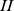). For TFBs, variations for different resistances in case 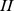 are generally more pronounced than case 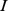 where, except for very small 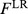, the distance among curves appears essentially proportional to the value of 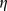, with smaller values contributing to smaller TFBs. For case 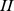, stochasticity accentuates the differences among curves, causing a quicker convergence to zero for lower resistance. Similarly to the case of Fig. 4, there exists an intersection point between case 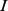 and 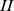 at lower 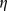, where, for sufficiently large tractions, persistent regimes are responsible for FBs happening at later times. This is also observed by comparing plots in the right column of Fig. 7, where, for 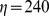 P (in red), the fraction of break-ups for case 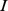 are larger than case 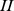 for 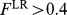 approximately. The message is clear: varying C-M sites is advantageous to stability only when resistance is sufficiently high, whereas, for smaller resistance, a persistent direction of motion allows a better preservation of pairs.

**Figure 8:**
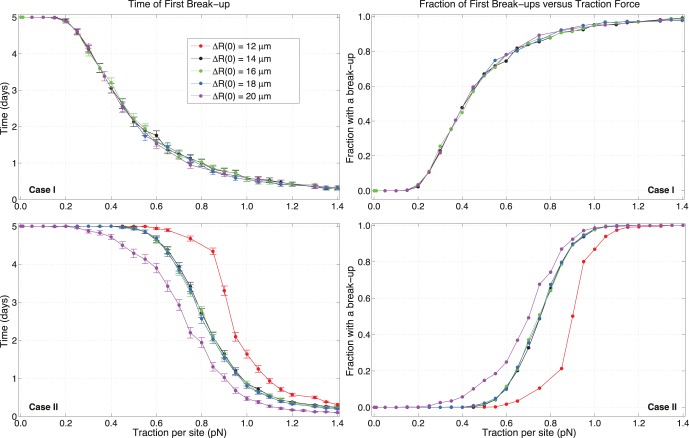
Role of initial distances on the fate of pairs. Times of first break-up and fractions of runs with a break-up versus traction per site strength 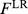, for different initial cell separations. Memory effects are present only when C-M sites are rearranged randomly at each 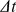.

Interestingly, active dynamic C-M sites seem to contribute to a memory effect that is not present for persistence, as illustrated in Fig. 8. In fact, when sites are static, the results are independent of whether cells start close to or far from each other, whereas the opposite occurs for case 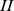. In particular, there is a notable difference between an initial bound state and a state where cells are only tangent. For a distance equal to almost half the diameter at time 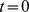 (in red in Fig. 8), cells experience a later FB and can sustain larger traction before a sensible fraction of pairs is broken. There appear to be three distinct regimes with diverse curves, depending on the initial distance. It is interesting that moderate overlaps at 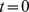 essentially share the same fate (in black, green and blue), whereas tangent cells (purple) and large overlaps have unique behavior (red). So, starting with larger adhesion due to a greater number of overlapping sites (see Eq.(5)) produces a stabilizing factor when stochasticity is present, but is irrelevant when C-M sites are static.

**Figure 9:**
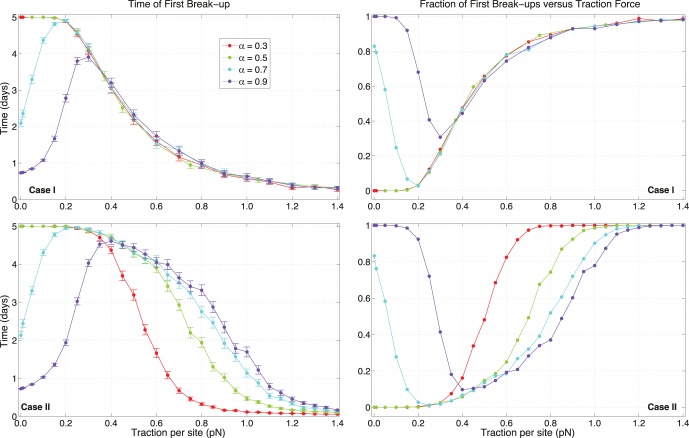
The effects due to cellular softness. Times of first break-up and fractions of runs with a break-up versus traction per site strength 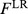, for different cellular softness values. When traction is fixed in time, i.e. case 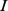, the rigidity of cells does not play any role and curves are identical, except for a non biological response at lower traction. For stochastic C-M sites, instead, pair stability increases with softness.

Analogously, the structural rigidity of cells plays a part only when C-M sites are randomly updated. In Fig. 9, results for different values of structural softness 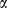 are collated. For sufficiently large tractions, cellular pairs in a persistent regime do not show any sensitively different behavior, both for the case of TFB and fractions of break-ups. At small 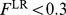, an effect similar to that shown in Fig. 4 is evident: when cells are very soft and adhesion is large, cells are forced to overlap for distances that are too small, and experience a rebound due to the infinite wall in Eq.(9). As previously remarked, this effect is not biologically realistic and has to be ignored. For case 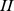, curves at different 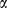 do not align for large tractions but maintain different profiles, indicating that the stability of pair dynamics does depend on cellular softness, with larger TFBs and fewer break-up events as stiffness decreases. In other words, adhesion is less effective when pairs are rigid, since their bound states occur at larger intercellular distances than those for softer pairs.

While ECM resistance produces variations in cellular interactions regardless of the motion of sites, dependence on initial separation between cells and cellular stiffness matter only if traction sites are dynamic.

### Effect of Depleting Adhesive Forces on Pair Dynamics

To complete our analysis, it is relevant to observe how a decrease in adhesive C-C site strengths can influence the behavior of cell pairs. This captures aspects of complex cellular programs where adhesion among receptors becomes less effective with time, as, for instance, in the epithelial-to-mesenchymal transition [59], [60].

For simplicity, we assume that the adhesion force acting between C-C sites is subject to a decrease in magnitude with time, and investigate changes in pair statistics as the rate of change of adhesion is varied. So, if 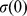 is a given initial adhesion strength, 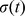 depends on a power function 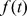:
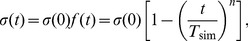
(10)where 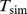 is the total simulation time for the run(s) and 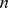 regulates the decay law. In the following, the values 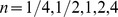 are considered, and cases 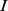 and 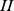 for batches of 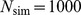 at a fixed 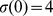
×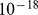 J are analyzed. Again, the total simulation time is 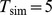 days, each simulation has a different, random distribution of sites at the initial 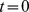 and 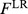 varies for a range of values.

**Figure 10:**
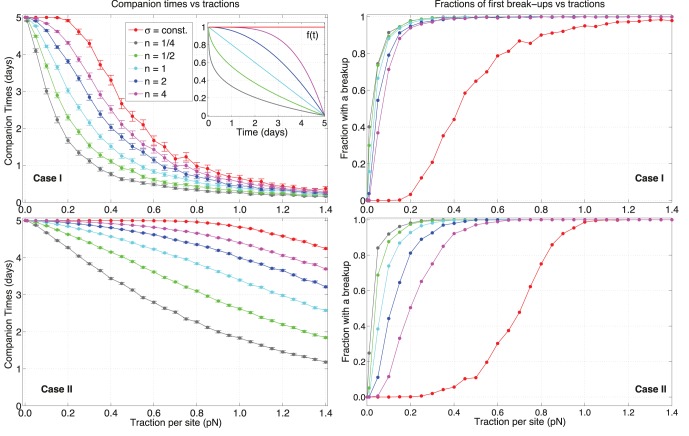
Effects of adhesion depletion for different decay laws. Comparison for companion times and fractions of break-ups versus traction per site strength 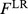 for cases 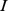 and 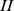. The inset subfigure shows the adhesion decay law 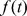. The colors depend on the exponent 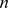. The case where adhesion is constant is in red. The initial value of adhesion is given by 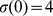
×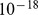 J. Note how stochastic traction forces are overall less sensitive than persistent pairs.

A depiction of 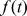 in Eq.(10) is given in the Fig. 10 inset, where a comparison of cases 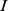 and 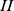 is shown. As expected, different decay laws operate differently on the CTs and fractions of break-ups for both cases. As 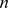 decreases, the depletion of adhesive force is more rapid, and the cell pair is less stable. For persistence (case 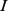), the effect of degradation on CTs is more relevant in the small to average range of tractions, where there is a net separation among curves at different 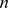. Instead, as 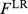 gets larger, all curves plateau at a similar value.

It is important to understand that, even if adhesion goes to zero for all 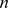 at 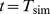, this does not imply that cells are always separated at the end of the simulation. In fact, for sufficiently low tractions, cells initially form a bound state and if the reduction in adhesion is not fast or 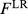 is not strong enough, then cells are not able to move apart with an adequate velocity. So, although their mutual distance is increasing with time, at 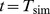 they are still linked. This is shown at 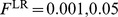 pN, where CTs are approximately equal to 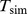 for almost every 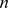 for both cases.

Looking at the fractions of pairs with a break-up for case 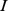 (top right Fig. 10), the impact of depletion of adhesion is evident. When compared with a constant 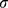, degradation causes a quick rise in the number of pairs that experience a break-up, and curves reach a constant value quite rapidly. Different decay laws display similar trends because, if traction is sufficiently high, pairs sooner or later separate regardless of how quick adhesion degrades. As expected, the higher the 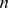 value the more stable the simulations are, since depletion is slower.

For case 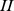 (bottom Fig. 10), there are some notable differences with respect to persistence. For the tractions investigated, CTs do not converge to low values like case 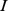. The effect of depletion on case 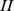 respect to case I is less dramatic. Compared with case 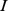, cells with dynamic C-M sites show larger CTs for each given 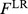. The separations among curves at different 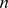 remain almost constant for 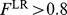. Moreover, the rise of curves in the bottom right plot is less abrupt than the case of persistence, and they are slower to asymptote to one. The larger the 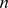 value, the larger is the difference between cases 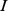 and 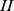 in the fractions with a break-up. This means that, the faster the depletion, the occurrence of break-ups depends less on C-M site dynamics.

In conclusion, the stability of pairs when adhesion force weakens in time is dependent on the dynamics of traction sites, but only if depletion is fast. If C-M sites do not move, cells separate sooner than for the case of constant adhesion, and a larger range of CTs than for case 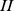 is possible. This is due to the traction being exerted in one fixed direction and the cells not being able to rejoin after a break-up occurs. On the other hand, motile sites generally increase the stability of pairs, and depletion has a less destabilizing effect on cells than in case 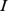.

### Simulation of Larger Cellular Assemblies

**Figure 11:**
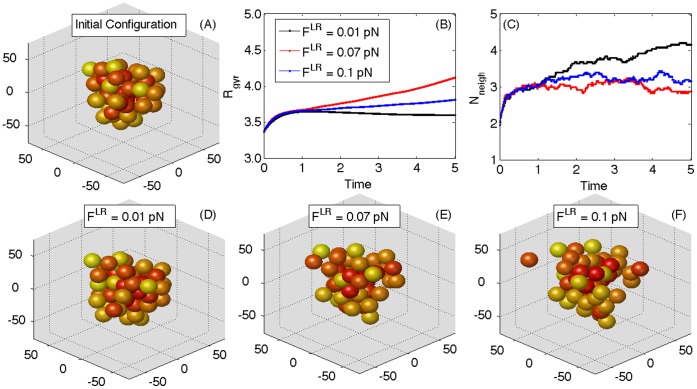
An example of the dynamics of a cluster of 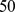 cells. From the same initial configuration (A), 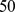 cells behave differently depending on the traction each cell exerts. Here 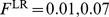 and 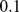 pN for each C-M site in each cell. Cellular positions are randomly assigned, with the constraint that no two overlapping cells enter each others’ impenetrable cores at 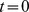. The distribution of C-C and C-M sites at the initial timestep is the same for each simulation run, and does not change with time. The structural compressibility is 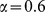 for all cells, and the adhesion per site is 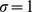
×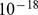 J. In (B) and (C), the evolution of the radius of gyration 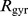 and the overlapping average number of cell neighbors per cell 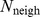 are displayed. The final configurations at 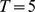 days are presented in (D), (E) and (F). The scale in the axes of all configuration plots (i.e, (A) and (D)–(F)) is 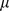
m.

As emphasized, our framework has been developed to understand better break-up mechanisms for large clusters of cells. As an example, results from single simulation runs for a collection of 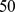 cells are presented. In Fig. 11(A), the initial cellular configuration at 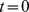 is shown, and the dynamics for a total time of 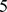 days are described using the radius of gyration of the cluster 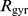 and the average number of overlapping neighboring cells 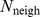 for each cell (Figs. 11(B)–(C)). The distribution of C-C and C-M sites is the same in all three realizations, and the traction per site assumes values 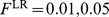 and 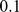 pN. These have not been reallocated with time (i.e., case 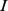 or persistent).

When a larger assembly of cell is considered, overlaps among more than two cells can be present. Recall that overlapping C-C sites obey Eq.(5), so that each interaction occurs between one site belonging to a cell 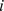 and another site belonging to a different cell 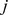. The same overlapping C-C site cannot simultaneously interact with two or more cells, because this would violate the one-to-one hypothesis employed in this model. If a given site is in the region of overlap of more than two cells, so that more than one cell offers a potential partner site for Eq.(5), the cell with which that site interacts is chosen at random for each 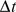.

Different cell-ECM tractions give rise to different dynamics, as the final configurations reached by the assembly at 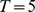 clearly show (see Figs. 11(D)–(F)). At this chosen, low value for C-C adhesion, i.e. 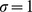
×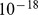 J, the smallest value 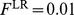 pN causes little or no movement to the cells. After a brief transient, there is an almost constant radius of gyration and an increasing 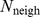, with minimal cellular rearrangement and no break-ups. At an average value of 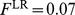 pN, the cluster expands and 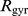 grows as a consequence of the increase in cellular mutual distances. Our definition of 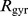 is not limited to an unbroken group of cells, but takes into account all the cells in the simulation run, regardless of them being still part of a common structure or moving freely. So, as cells detach and get away from each other with a constant velocity, 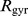 grows linearly. At the largest value of tractions, 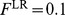 pN, the disaggregation of the cluster is more rapid and more cells display break-ups. The remaining group of cells are smaller than the case of 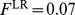 pN, and cells organize in couples and triplets (Fig. 11(F)), with some cells completely detached and travelling alone through the ECM. The rate of growth of 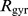 is steepest for 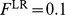 pN, because cells in isolation move faster when 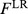 is larger. Fig. 11(C) shows that 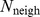, after an initial increasing phase, tends to stabilize at values inversely proportional to the traction rates. For larger values of C-C adhesions, for example 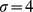
×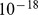 J (not shown), the cluster tends to contract and can withstand larger tractions, showing no detachments of cells for the same ranges of 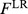 shown in Fig. 11.

## Conclusions

A new and general framework for the study of three-dimensional, collective cellular motility in the extracellular matrix has been developed and trialled. The model is off-lattice, its parameters are experimentally accessible and it presents a defined focus on the receptors that mediate cell-cell and cell-ECM interactions. As a relevant case study and to illustrate the capabilities of this framework, the behavior of two cells has been extensively analyzed, under a variety of situations, capturing relevant changes in cellular individual properties and ECM parameters.

It has been shown that the motility of C-M receptors and the stochasticity of traction forces, depending on the magnitude of cell-cell adhesion, can increase the overall time cells spend together. When this occurs, cells tend to aggregate and form stronger bonds, and, if they detach, they show a more pronounced ability to rejoin nearby companions. On the contrary, the model shows that static C-M contacts discourage such behaviors.

The role of adhesion and traction sites has been particularly emphasized, analysing different scenarios of coupled and uncoupled motility. The stability of pairs can change widely according to model parameters, and, in particular, interactions between the forces mediated by adhesion and traction sites appears crucial. For instance, cases have been shown where a stronger adhesion is not advantageous unless traction is sufficiently strong, or motility of cell-cell sites have opposite effects on pair stability according to the strength of cell-ECM traction. This interdependence between traction and adhesion forces is important, and clearly shows the delicate role that cellular receptors play in the dynamics.

Break-up events can occur at different times and with different frequencies, and strongly depend on the regimes considered. The two types of receptors contribute to the overall stability of pairs in unique ways, and variations of C-C and C-M sites together or alone make observable differences in the fate of pairs. Again, the impact of site motion on cellular behaviors depends on the magnitude of adhesion and traction forces. Generally, it emerges that small changes in the parameters can strongly affect the interplay among the forces, resulting in a great variability in the final cellular state.

The way the initial intercellular distance and cellular rigidity influence motility is related to the stochasticity of sites. Memory effects are present when C-M receptors change their positions at each timestep. On the contrary, resistance to the ECM has been shown to change the behavior of two cells independently of site dynamics, with larger effects in the case of randomness.

When traction is pointed towards a fixed direction, break-up events are generally irreversible and there are very few chances that pairs can rejoin at later times. This phenomenon has been illustrated in detail. For the case of random motion of sites, we have found that there exists an optimal compromise between forces: after a break-up event, the companion time is maximal for values of traction force that are dependent on the mutual adhesion. At those values, cells rejoin more efficiently and for longer durations, and generally travel together for longer distances. This is another interesting result, where it clearly appears how overall motility depends on either cell-cell and cell-ECM interactions.

In conclusion, our model provides us with a framework to analyze quantitatively the influence of cell-cell and cell-matrix interactions and cell-matrix interfacial forces on collective motion in 3D. We believe that it is able to provide researchers in a spectrum of disciplines with a quantitatively rigorous framework to study more complex dynamic cellular and multi-cellular processes in native-like in vitro and in vivo environments.
